# LncRNA *EN-90756* promotes CPB2-induced proliferation and inhibits apoptosis in IPEC-J2 cells by affecting the JAK-STAT signaling pathway activation

**DOI:** 10.3389/fmicb.2022.1082025

**Published:** 2023-01-12

**Authors:** Jiaojiao Yang, Juanli Zhang, Qiaoli Yang, Xiaoyu Huang, Zunqiang Yan, Pengfei Wang, Xiaoli Gao, Jie Li, Na Li, Yi Gao, Shuangbao Gun

**Affiliations:** ^1^College of Animal Science and Technology, Gansu Agricultural University, Lanzhou, China; ^2^College of Life Sciences and Technology, Longdong University, Qingyang, China; ^3^Jilin Rongtai Agricultural Development Co., Ltd., Changchun, China; ^4^Gansu Research Center for Swine Production Engineering and Technology, Lanzhou, China

**Keywords:** RNA-seq, lncRNAs, IPEC-J2, CPB2 toxin, piglet diarrhea

## Abstract

**Background:**

Long non-coding RNAs (lncRNAs), as key regulators, are closely associated with the development of a variety of disease. However, the mechanisms by which lncRNAs regulate *Clostridium perfringens* type C induced piglet diarrhea are unclear.

**Methods:**

In the present study, we explored the expression and characterization of lncRNAs in a *C. perfringens* beta2 (CPB2) toxin-treated intestinal porcine epithelial cell line-J2 (IPEC-J2) using RNA-sequencing (RNA-seq).

**Results:**

A total of 6,558 lncRNAs were identified, of which 49 lncRNAs were significantly differentially expressed between the control and CPB2 groups. Functional enrichment analysis showed that the target genes of differentially expressed lncRNA *EN-90756* were mainly associated with defense response to virus, and negative regulation of apoptotic process. LncRNA *EN-90756* was significantly up-regulated in IPEC-J2 cells at different time points after CPB2 treatment. Functionally, knockdown of lncRNA *EN-90756* might regulate the proliferation and apoptosis of IPEC-J2 cells by affecting the Janus kinase (JAK)-signal transducer and activator of transcription (STAT) signaling pathway. LncRNA *EN-90756* may be involved in CPB2 toxin-induced piglet diarrhea by regulating the expression of its target gene *MX1* (encoding MX dynamin like GTPase 1).

**Conclusion:**

Long non-coding RNA *EN-90756* affected the antiviral ability of IPEC-J2 cells by regulating the expression of MX1. Meanwhile, lncRNA *EN-90756* might regulate cell proliferation and apoptosis by affecting JAK-STAT signaling pathway activation. These findings provide novel perspectives and directions for further exploration of the regulatory mechanisms of lncRNAs on CPB2 toxin-induced diarrhea in piglets.

## 1. Introduction

Piglet diarrhea has a high incidence and brings huge economic losses to pig farms (Silva et al., 2015). *Clostridium perfringens* type C is one of the main pathogens causing diarrhea in piglets, producing *C. perfringens* beta2 (CPB2) toxin that acts directly or indirectly on the intestines, causing intestinal damage and triggering an inflammatory response (Zeng et al., 2016). Several epidemiological studies suggested that CPB2 toxins are highly associated with intestinal diseases in domestic animals. NetB toxin and CPB2 toxin produced by *C. perfringens* are associated with subclinical necrotizing enteritis in laying hens (Timoney et al., 2005). CPB2 toxin might play a role in the pathogenesis of enterotoxaemia in calves (Lebrun et al., 2007). There is a significant association between CPB2-positive *C. perfringens* isolates and piglet diarrhea (Waters et al., 2003). CPB2 toxin is significantly cytotoxic to intestinal porcine epithelial cell line-J2 (IPEC-J2) cells inhibiting cell growth and increasing cell permeability in a concentration-dependent manner (Luo et al., 2020). CPB2 toxin induces apoptosis and increases inflammatory markers in IPEC-J2 cells and impairs intestinal barrier function (Gao et al., 2020).

Long non-coding RNAs (lncRNAs) are RNAs comprising more than 200 nucleotides that have almost no protein-coding capacity and play key roles in a variety of biological processes, including epigenetic regulation, transcriptional regulation, metabolism, and immune responses (Marzia et al., 2018). In recent years, studies have reported that lncRNAs have important functions in piglet resistance to pathogenic infections causing diarrhea. Down-regulation of lncRNA *FUT3-AS1* expression contributed to enhancing the resistance of IPEC-J2 cells to *Escherichia coli* F18 infection (Wu et al., 2022). Porcine endemic diarrhea virus (PEDV) infection modulates lncRNA expression patterns in the ileum of IPEC-J2 cell lines and piglets and activates the ileal immune system (Chen et al., 2019). In piglet diarrhea caused by *C. perfringens* type C, many lncRNAs and mRNAs modulate drug resistance and susceptibility in piglets through immune-related pathways (Huang et al., 2019). Although many lncRNAs have been reported to be associated pig diarrhea, few lncRNAs have been identified that are closely associated with CPB2 toxin-induced diarrhea in piglets.

Our previous study used CPB2 toxin (20 μg/ml) to treat IPEC-J2 cells for 24 h to construct an *in vitro* model of *C. perfringens* type C infected piglet diarrhea (Gao et al., 2020). In this study, we used RNA sequencing (RNA-seq) to perform transcriptome analysis of the CPB2 and control groups of IPEC-J2 cells and to identify differentially expressed lncRNAs in response to CBP2. We also analyzed the gene ontology (GO) enrichment of differential lncRNAs and carried out Kyoto Encyclopedia of Genes and Genomes (KEGG) pathway analysis with the aim of identifying possible molecular mechanisms of CPB2 toxin-induced diarrhea in piglets. We identified a key lncRNA, lncRNA *EN-90756*, which was highly expressed in CPB2-induced IPEC-J2 cells and applied a series of biological experiments to explore its regulatory mechanism. These results increase our understanding of the molecular mechanisms of lncRNAs in CPB2 toxin-induced diarrhea in piglets.

## 2. Materials and methods

### 2.1. Preparation and purification of the CPB2 toxin

The CPB2 toxin was extracted and purified according to Luo’s method (Luo et al., 2020). Briefly, a recombinant plasmid containing the CPB2 gene, pET-28a-CPB2, was constructed transformed into BL21 *E. coli* cells for expression. The expressed CPB2 protein was purified using High Affinity Ni-Charged Resin FF (GenScript, Nanjing, China) and concentrated using PEG6000. Finally, a ToxOut™ Rapid Endotoxin Removal Kit (AmyJet Scientific, Wuhan, China) was used to remove the endotoxin.

### 2.2. Cell culture and processing

Intestinal porcine epithelial cell line-J2 cells were provided by the BeNa Culture Collection (Beijing, China). Cells were cultured using Dulbecco’s modified Eagle’s medium (HyClone, Logan, UT, USA) containing 10% fetal bovine serum (Gibco, Grand Island, NY, USA) and 1% double antibodies (penicillin and streptomycin) at 37°C in an atmosphere of 5% CO_2_. The CPB2 group of cells was treated with the CPB2 toxin (20 μg/ml). Three replicates were set up for each group.

### 2.3. RNA-sequencing (RNA-seq) and data analysis

Total RNA was extracted from the control and CPB2-treated cells using RNAiso™ Reagent (Invitrogen, Carlsbad, CA, USA). The total RNA quantity and purity were assessed using a Bioanalyzer 2100 and RNA 1000 Nano LabChip Kit (Agilent, Santa Clara, CA, USA), respectively. The RNA samples were of high quality with RNA integrity numbers > 7. Ribosomal RNA was removed using a Ribo-Zero™ rRNA Removal Kit (Illumina, San Diego, CA, USA) and then reverse transcribed into cDNA. The cDNA libraries were constructed and sequenced on an Illumina NovaSeq™ 6000 platform (Illumina, San Diego, CA, USA) using 2 × 150 bp paired-end sequencing. The raw data obtained from IPEC-J2 samples were stored in the NCBI SRA database (accession number: PRJNA749943).

The raw data were processed using FastQC software.^1^ This step produced clean data by removing the reads with shifted connectors and low quality sequences (quality score < Q30). The reads were mapped against the pig genome assembly (*Sscrofa* 11.1) and the alignment results were assessed using HISAT2^2^ (Kim et al., 2015). StringTie software^3^ was used to estimate gene expression levels according to fragments per kilobase of transcript per million mapped reads (FPKM) (Trapnell et al., 2010). After all transcripts were obtained, transcripts smaller than 200 bp and known mRNAs were removed and lncRNA prediction was performed on the remaining transcripts using CPC, Pfam-sca, and CNCI (Kong et al., 2007; Sun et al., 2013). LncRNAs with fold change (FC) ≥ 2 and *p* < 0.05 were regarded as significantly differentially expressed.

Long non-coding RNA target genes were predicted using both *cis* and *trans* methods. LncRNA *cis*-regulatory target genes were mainly predicted based on positional relationships. The mRNAs that were differentially expressed within 100 kb upstream and downstream of the lncRNA were considered to be lncRNA *cis*-regulatory target genes. The *trans*-regulated target genes of lncRNAs were analyzed using RIsearch (Wenzel et al., 2012) software. Screening conditions included the formation of secondary structure free energy between the lncRNA and mRNA sequences (energy < −11) and the Pearson correlation coefficient (*r* > 0.95 or *r* < −0.95). Subsequently, GOSeq Release 2.12 and KO-BAS (V2.0) (Kanehisa et al., 2007; Young et al., 2010) were used to perform GO and KEGG pathway enrichment analyses of the target genes of the lncRNAs, respectively.

### 2.4. Quantitative real-time reverse transcription polymerase chain reaction (qRT-PCR)

Total RNA was extracted from IPEC-J2 cells (1 × 10^7^) according to the method described in section “2.3 RNA-sequencing (RNA-seq) and data analysis.” Cytoplasmic and nuclear RNAs of IPEC-J2 cells were isolated using a PARIS™ Kit reagent kit (Ambion, Austin, TX, USA) according to the instructions provided by the supplier. Total RNA was reverse transcribed into cDNA using Evo M-MLV Mix Kit with gDNA Clean for qPCR AG11728 (Accurate Biotechnology, Hunan, China). The qRT-PCR assay was performed according to the instructions of SYBR Green assay (Accurate Biotechnology, Hunan, China) and performed on a Roche LightCycler 480 (Roche Applied Science, Mannheim, Germany). Gene expression levels were normalized to that encoding glyceraldehyde-3-phosphate dehydrogenase (*GAPDH*) to determine the relative expression using the 2^–ΔΔCt^ method (Ljvak, 2001). The PCR primers for transcripts and genes are listed in Table 1.

**TABLE 1:** Quantitative real-time reverse transcription polymerase chain reaction (qRT-PCR) primers for amplifying specific genes.

GeneID/Gene name		Nucleotide sequence (5′–3′)	Product length (bp)
*ENSSSCG00000041190*	Forward	GAATCTGAACTTCTTGCCACA	112
	Reverse	TACTAACATTCCCTGCCCAT	
*ENSSSCG00000047438*	Forward	CATTATGTGCCACCGACGAA	178
	Reverse	TCACTGAAAGCCAAGTGTC	
*ENSSSCG00000046898*	Forward	TTTATTTGGAAGCGGACCCT	110
	Reverse	TCCCCTCATTCTCAGAGTCG	
*ENSSSCG00000045823*	Forward	CCTTGCCTCACTCGGTGT	239
	Reverse	GGATCCTATGCAGCAACCC	
*LOC106507243*	Forward	CGGCGCTTCACAGACTCC	143
	Reverse	GGCTCCAGGAACAAGTACCC	
*LOC102162336*	Forward	GCCCATCCCTTGAAAATGACGA	157
	Reverse	CCGCCCTTCCTCAACACC	
*ENSSSCG00000041166*	Forward	GAAACGAATCTGACTAGCACCC	165
	Reverse	TAATCCCTGGCCTCACTCGG	
*ENSSSCG00000047080*	Forward	CCCCGACCTGATAAGTCCT	105
	Reverse	AGGCCATCTTCCCTAATCCCT	
*LOC106508476*	Forward	GGAAACAGCCTAAATGGTCA	160
	Reverse	GGCCTCATGTAACAAGGACTCA	
*ENSSSCG00000048701*	Forward	AGACTCTGACGTGGTAGGACA	145
	Reverse	TTGGAGAAGTGTCACACCGT	
*U6*	Forward	TTATGGGTCCTAGCCTGAC	224
	Reverse	CACTATTGCGGGTCTGC	
*MX1*	Forward	CCACCTGAAGAAGGGCTAC	219
	Reverse	AACAGGGGCAGAGTTTTAC	
*GAPDH*	Forward	AGTATGATTCCACCCACGGC	139
	Reverse	TACGTAGCACCAGCATCACC	
*Caspase 3*	Forward	CCGAAATGTTTGCTGACGGC	152
	Reverse	CCGATCTCGAAGGAAGTCCA	
*Caspase 8*	Forward	CGGCTCTGAGCAAGACCTTTA	173
	Reverse	GCCGTAGATGATGCCCTTGT	

### 2.5. Single RNA fluorescent *in situ* hybridization (FISH)

The green fluorescent FAM-labeled probe for lncRNA *EN-9075* (lncRNA-FISH probe mix) was designed by GenePharma (Shanghai, China) and detected using a fluorescent *in situ* hybridization kit (GenePharma) according to the manufacturer’s instructions. In short, IPEC-J2 cells were inoculated overnight in 48-well plates at a density of 1 × 10^4^ cells/well and then treated with CPB2 toxin for 24 h. IPEC-J2 cells were then fixed with 4% (v/v) paraformaldehyde for 15 min at room temperature. The IPEC-J2 cells permeabilized in phosphate-buffered saline (PBS) containing 0.1% Triton X-100 for 15 min at room temperature and then blocked with pre-hybridization buffer for 30 min at 37°C. The IPEC-J2 cells were treated with the FAM-labeled probe in hybridization buffer (10 μL, 10 μM probe, and 90 μL hybridization buffer) the dark at 37°C for 14 h. The cells were then washed three times with wash buffer (4 × SSC/2 × SSC) in the dark and stained with 4′, 6-diamidino-2-phenylindole for 15 min. The IPEC-J2 cells were washed three times with PBS and then observed under a fluorescent microscope at 200× magnification (Olympus IX71, Tokyo, Japan).

### 2.6. Cell transfection

The small interfering RNA targeting lncRNA *EN-90756* (si EN-90756) and the negative control siRNA (si NC) were obtained from GenePharma. IPEC-J2 cells (1 × 10^5^ cells/ml) were grown overnight in six-well plates until they were 70% confluent for transfection. Lipofectamine 2000 (Invitrogen) was mixed with Opti-MEM medium (Invitrogen, CA, USA) and left for 5 min. Then, the solution was added to si EN-90756, si NC (1:1 ratio) diluted with Opti-MEM medium, mixed and left for 20 min. The prepared mixtures were added to IPEC-J2 cells separately and transfection was completed after 24 h.

### 2.7. Cell viability and proliferation assay

Intestinal porcine epithelial cell line-J2 (density of 5 × 10^3^/well) were seeded in 96-well plates, with three replicates for each group. After treatment with CPB2 toxin, cell viability was determined according to the instructions of Cell Counting Kit-8 (Beyotime, Shanghai, China). IPEC-J2 cells (1 × 10^5^ cells/ml) were grown in six-well plates and treated with CPB2 toxin or PBS. Cell proliferation was assayed using the BeyoClick™ EdU Cell Proliferation Kit with Alexa Fluor 488 [5-ethynyl-2′-deoxyuridine (EdU), Beyotime]. Cell proliferation was observed under a fluorescence microscope at 200× magnification (Olympus IX71).

### 2.8. Flow cytometry

Intestinal porcine epithelial cell line-J2 cells (1 × 10^5^ cells/ml) were grown in six-well plates, transfected with si NC or si EN-90756 for 24 h and then treated with CPB2 toxin for 24 h. Cells were collected and washed twice with PBS. Then, 1 ml of pre-cooled 70% ethanol was added to the cells, mixed with gentle shaking, and fixed at 4°C for 12 h. The ethanol was discarded, the cells were washed using PBS and collected by centrifugation. Then, the cells were incubated with Annexin V-fluorescein isothiocyanate and propidium iodide for 20 min at 25°C in the dark. Apoptosis was detected using a FACSCalibur flow cytometer (BD Biosciences, San Jose, CA, USA).

### 2.9. Mitochondrial membrane potential assay

Intestinal porcine epithelial cell line-J2 cells were transfected with si NC or si EN-90756 and treated with CPB2 toxin or PBS and then assayed for mitochondrial membrane potential (Δψm) according to the instructions of the mitochondrial membrane potential assay kit with JC-1 (Beyotime). Briefly, cells were incubated with JC-1 staining solution at 37°C for 20 min, washed two times with JC-1 staining buffer (1×) and then observed under a fluorescent microscope (Olympus IX71).

### 2.10. Western blotting

Total IPEC-J2 cell proteins were extracted using Radioimmunoprecipitation assay (Beyotime). The extracted proteins were quantified using a bicinchoninic acid protein analysis kit (Beyotime). Sodium dodecyl sulfate-polyacrylamide gel electrophoresis (12%) was used to separate equal amounts of proteins, which were then transferred to polyvinylidene fluoride membranes (Millipore, Billerica, MA, USA). The membranes were incubated with primary antibodies overnight at 4°C.

The primary antibodies comprised those recognizing: MX dynamin like GTPase 1 (MX1) (bsm-51528m, Bioss, Woburn, MA, USA; 1:1,000); Janus kinase 1 (JAK1) (bs-1439R, Bioss; 1:1,000); phosphorylated (p-JAK1) (bs-3238R, Bioss; 1:1,000); signal transducer and activator of transcription 3 (STAT3) (GB11176, Servicebio, Wuhan, China; 1:1,000); p-STAT3 (bs-1658R, Bioss; 1:1,000); and GAPDH (GB15002, Servicebio; 1:2,000). After incubation with horseradish peroxidase-labeled secondary antibodies (GB23303, Servicebio; 1:5,000) or (GB25301, Servicebio; 1:5,000), the immunoreactive protein bands were observed using the enhanced chemiluminescence method (Thermo Fisher Scientific, Waltham, MA, USA).

### 2.11. Statistical analysis

The results were statistically analyzed using SPSS v.21 software (IBM Corp., Armonk, NY, USA). All experiments had three replicates, and the experimental data are expressed as the mean ± standard deviation (SD). Differences were determined using Student’s *t*-test (two-tailed test). *P*-values < 0.05 were considered statistically significant.

## 3. Results

### 3.1. Quality analysis of the RNA-seq data

**FIGURE 1:**
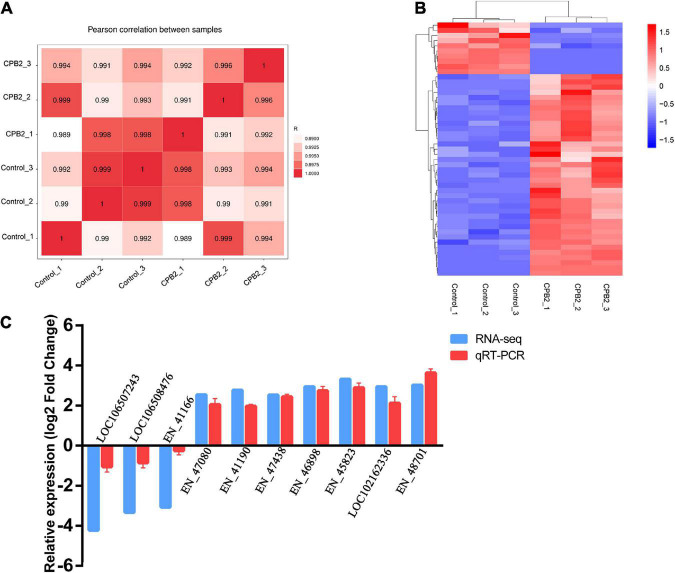
Quality analysis of the RNA-seq data. **(A)** Chart of the expression correlation between samples. *R* is Pearson’s correlation coefficient. **(B)** A hierarchical heat map of differential long non-coding RNAs (lncRNAs) in the control and toxin-treated intestinal porcine epithelial cell line-J2 (IPEC-J2) cells. Data are expressed as the fragments per kilobase of transcript per million mapped reads. Different colors indicate different levels of gene expression, with colors ranging from blue through white to red indicating low to high expression. **(C)** Quantitative real-time reverse transcription polymerase chain reaction (qRT-PCR) validation of 10 differentially expressed lncRNAs.

Six cDNA libraries of IPEC-J2 cells from the control and CPB2 groups were sequenced and Pearson’s correlation coefficient between the two groups ranged from 0.98 to 0.99, indicating a high degree of similarity in expression patterns between the samples from the same group (Figure 1A). After quality control of the raw data, we obtained approximately 8.36 Gb of high quality data and the average GC content of the six libraries was approximately 53%; and the Q30 (sequencing error rate less than 0.001) per sample ranged from 95.34 to 96.14% (Supplementary Table 1). Over 70% of the clean reads were mapped to the porcine reference genome, containing approximately 60% of the unique mappings (Supplementary Table 2).

### 3.2. Identification of differentially expressed lncRNAs

A total of 6,558 lncRNAs were identified in IPEC-J2 cells in the control and CPB2 groups, among which 39 were significantly up-regulated and 10 were significantly down-regulated lncRNAs between the two groups of IPEC-J2 cells [*p*-value < 0.05 and | (log2 (FC)| ≥ 1)] (Figure 1B and Supplementary Table 3).

Ten significantly differentially expressed lncRNAs were randomly selected for qRT-PCR validation of the accuracy of the sequencing results. The expression levels of lncRNAs *LOC106507243*, *LOC106508476*, and *EN-41166* were down-regulated after CPB2 treatment, while lncRNAs *EN-47080*, *EN-41190*, *EN-47438*, *EN-46898*, *EN-45823*, *LOC102162336*, and *EN-48701* were up-regulated after CPB2 treatment (Figure 1C). The results of qRT-PCR were consistent with the RNA-seq results, demonstrating that the sequencing results were reliable and reproducible.

### 3.3. GO terms and KEGG pathways analysis of lncRNA *EN-90756* target gene

**FIGURE 2:**
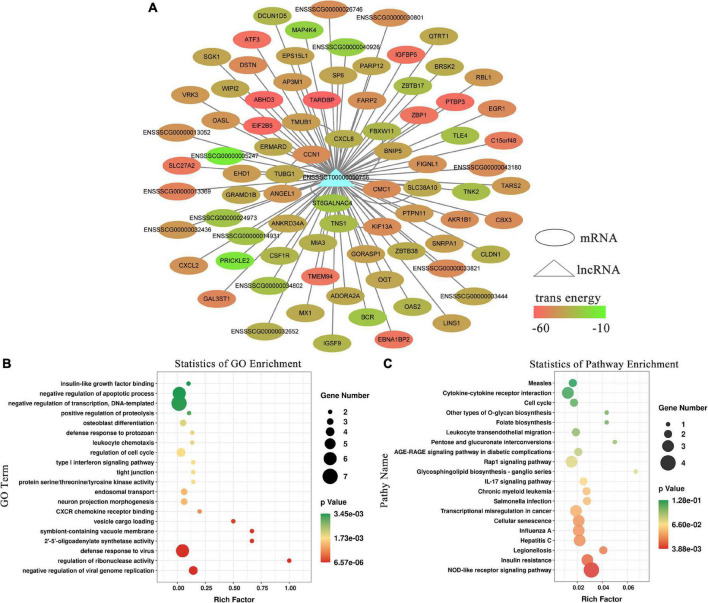
Functional enrichment analysis of long non-coding RNA (lncRNA) *EN-90756*. **(A)** Network between lncRNA *EN-90756* and differentially expressed mRNAs. The ellipse represents mRNAs, the triangle represents the lncRNA, and the color of the ellipse represents the formation of secondary structure free energy between the lncRNA and mRNA sequences. Functional enrichment analysis of lncRNA EN-90756 significantly differentially expressed target genes. **(B)** The top 20 significant gene ontology (GO) enrichment terms. **(C)** The top 20 enriched Kyoto Encyclopedia of Genes and Genomes (KEGG) pathways. In the scatter plot, the size of the dots represents the number of genes with significant differences, and the color of the dots represents the significance of the enrichment (*p*-value).

To explore their functions, the 49 differentially expressed lncRNAs were subjected to target gene prediction. Consequently, genes associated with the immune response and viral defense (e.g., *CXCL2*, *MX1*, OASL) were identified among the target genes (Figure 2A) of the significantly differentially expressed lncRNA *EN-90756*. Further enrichment analysis revealed that significantly enriched GO terms were defense response to virus, negative regulation of viral genome replication, negative regulation of apoptotic process, and regulation of cell cycle (Figure 2B). The KEGG enrichment analysis identified Legionellosis, influenza A, Salmonella infection, and cellular senescence signaling pathways were significantly enriched (Figure 2C). Interestingly, the *MX1* gene was enriched in the defense response to virus, cellular response to type I interferon, and hepatitis C, influenza A, and measles signaling pathways. These results suggest that lncRNA *EN_90756* might be involved in the invasion of IPEC-J2 cells by CPB2 toxin through the regulation of target genes.

### 3.4. LncRNA *EN-90756* was highly expressed in CPB2-treated IPEC-J2 cells

**FIGURE 3:**
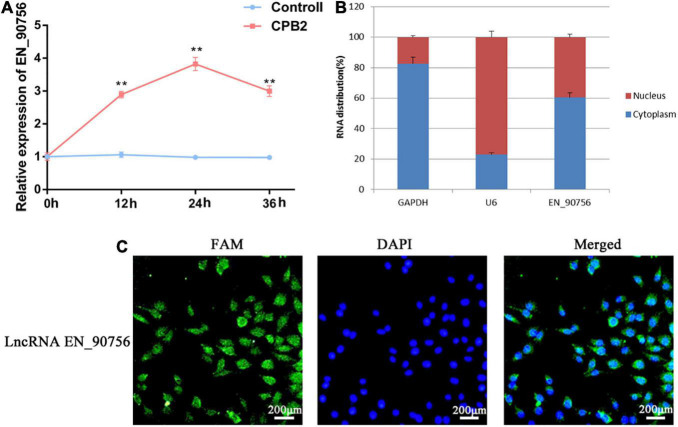
*Clostridium perfringens* beta2 (CPB2) toxin induction promotes high expression of long non-coding RNA (lncRNA) *EN-90756* in intestinal porcine epithelial cell line-J2 (IPEC-J2) cells. **(A)** Changes in lncRNA *EN-90756* expression from 0 to 36 h in IPEC-J2 cells treated with 20 μg/ml CPB2. **(B)** Nuclear/cytoplasmic isolation showed the distribution of lncRNA EN-90756 in the cells. **(C)** Fluorescent *in situ* hybridization (FISH) analysis of IPEC-J2 cells treated with CPB2 for 24 h using a FAM-labeled lncRNA EN-9075 probe (green) and 4′, 6-diamidino-2-phenylindole nuclear staining (blue). Scale bar = 200 μm. **p < 0.05.

The expression pattern of lncRNA *EN-90756* in CPB2-treated cells was determined at different time points (0, 12, 24, and 36 h) of CPB2 toxin treatment. The results showed that lncRNA *EN-90756* expression was significantly up-regulated during CPB2 toxin treatment and reached its highest level at 24 h (Figure 3A). The nuclear/cytosol separation assay showed that lncRNA *EN-90756* was mainly located in the cytoplasm, with a small amount in the nucleus (Figure 3B). RNA-FISH results also confirmed that the green fluorescent signal of lncRNA *EN-90756* was distributed in both the nucleus and the cytoplasm, but was mainly located in the cytoplasm (Figure 3C). These results suggest that lncRNA *EN-90756* might be an important regulator in the regulation of *C. perfringens* type C infection.

### 3.5. Knockdown of lncRNA *EN-90756* inhibited the proliferation of IPEC-J2 cells

**FIGURE 4:**
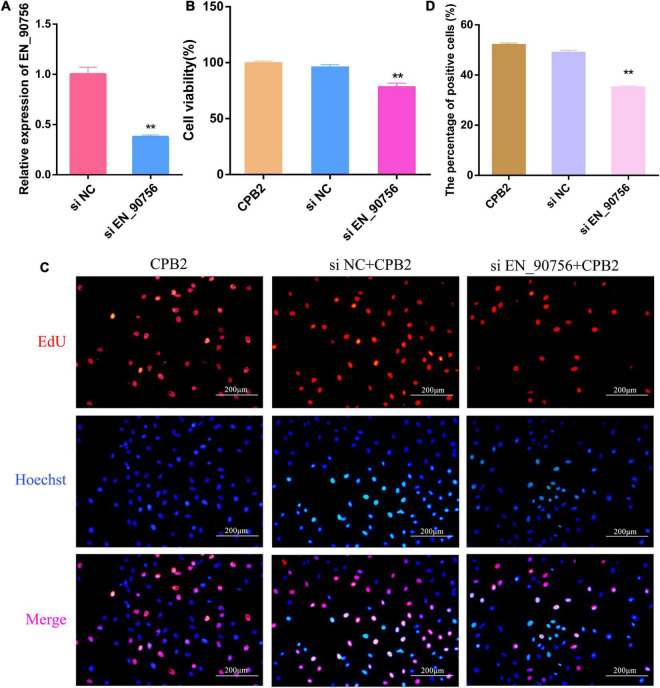
Knockdown of long non-coding RNA (lncRNA) *EN-90756* inhibits the proliferation of intestinal porcine epithelial cell line-J2 (IPEC-J2) cells. **(A)** Expression of lncRNA EN-90756 in *Clostridium perfringens* beta2 (CPB2) cells after transfection with si NC and si EN_90756. **(B)** Effect of lncRNA EN-90756 on the viability of CPB2-treated IPEC-J2 cells. **(C)** si NC or si *EN_90756* were transfected into CPB2-treated IPEC-J2 cells, followed by staining with 5-ethynyl-2′-deoxyuridine (EdU) (red) for DNA replication and Hoechst (blue) for nuclei. Scale bar = 200 μm. **(D)** The percentages of EdU-positive cells. **p < 0.05.

Long non-coding RNA *EN-90756* was knocked down to investigate its role in CPB2-treated IPEC-J2 cells. The expression level of lncRNA *EN-90756* was significantly reduced after transfection with si EN-90756 (Figure 4A). Cell viability assays showed that knockdown of lncRNA *EN-90756* in CPB2-treated IPEC-J2 cells significantly reduced cell viability (to 78.75%) (Figure 4B). EdU analysis revealed that more EdU-positive cells were observed in the CPB2 and si NC + CPB2 groups, while fewer EdU-positive cells were observed in the si EN_90756 + CPB2 group (Figures 4C, D). These results indicated that down-regulation of lncRNA *EN-90756* inhibited the proliferation of IPEC-J2 cells.

### 3.6. Knockdown of lncRNA *EN-90756* promoted apoptosis in IPEC-J2 cells

**FIGURE 5:**
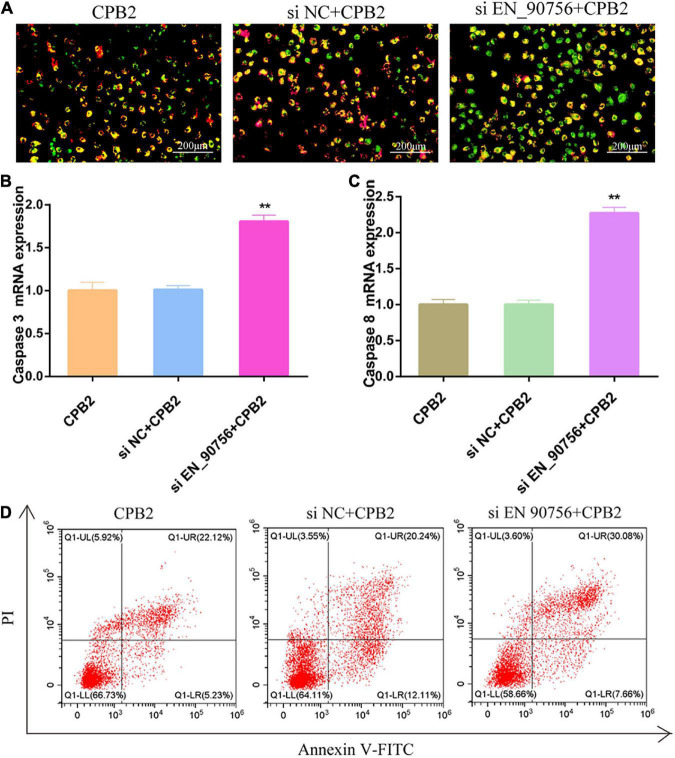
Knockdown of long non-coding RNA (lncRNA) *EN-90756* promoted apoptosis in intestinal porcine epithelial cell line-J2 (IPEC-J2) cells. **(A)** The Δψm was detected in IPEC-J2 cells treated with *Clostridium perfringens* beta2 (CPB2) for 24 h and stained using the JC-1 probe. Scale bar = 200 μm. The shift from red to green fluorescence represents a decrease in cell membrane potential. The mRNA expression of *caspase 3* **(B)** and *caspase 8* **(C)** in cells treated with 20 μg/ml CPB2 toxin for 24 h. **(D)** Apoptosis was detected using Annexin V-fluorescein isothiocyanate. **p < 0.05.

JC-1 staining showed that after knocking down lncRNA *EN-90756*, the fluorescence turned almost entirely green in CPB2-treated IPEC-J2 cells, reflecting the collapse of the Δψm (Figure 5A). This suggested that knockdown of lncRNA *EN-90756* exacerbated CPB2-induced loss of Δψm in IPEC-J2 cells. Caspase 3 and Caspase 8, are key proteins in apoptosis, and qRT-PCR revealed that the mRNA expression levels of *Caspase 3* and *Caspase 8* were significantly elevated in CPB2-induced IPEC-J2 cells after knockdown of lncRNA *EN-90756* (Figures 5B, C). Furthermore, the results of flow cytometry showed that the apoptosis rate was 27.35% in the CPB2 group and 37.74% in the si EN_90756 + CPB2 group (Figure 5D). These results suggested that down-regulation of lncRNA *EN-90756* promoted apoptosis in IPEC-J2 cells.

### 3.7. Knockdown of lncRNA *EN-90756* promoted MX1 expression and inhibited the JAK-STAT signaling pathway

**FIGURE 6:**
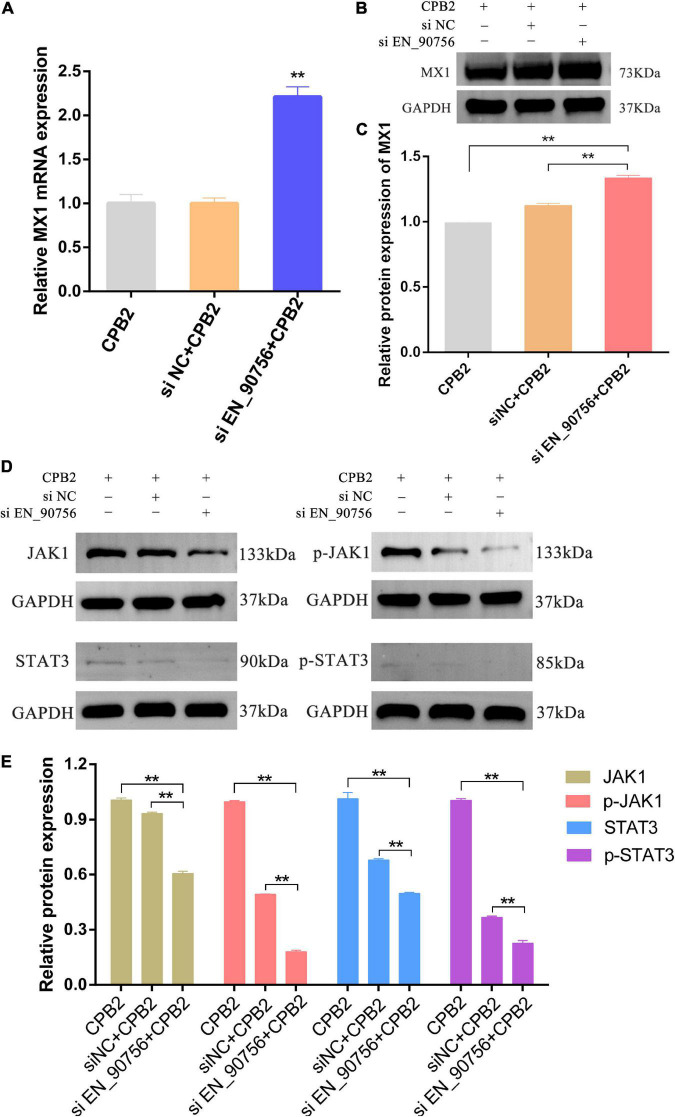
Inhibition of long non-coding RNA (lncRNA) *EN-90756* promoted MX1 expression and suppressed the Janus kinase (JAK)-signal transducer and activator of transcription (STAT) signaling pathway. The mRNA expression **(A)** and protein levels **(B,C)** of the target gene MX1 in *Clostridium perfringens* beta2 (CPB2)-induced intestinal porcine epithelial cell line-J2 (IPEC-J2) cells before and after lncRNA EN-90756 inhibition. **(D,E)** Western blotting analysis of JAK1, p-JAK1, STAT3, and p-STAT3 protein levels in IPEC-J2 cells after CPB2 stimulation by lncRNA *EN-90756* inhibition. **p < 0.05.

Long non-coding RNA *EN-90756* knockdown resulted in significant up-regulation of the expression of target gene *MX1* (Figure 6A) and its protein level (Figures 6B, C) in CPB2-induced IPEC-J2 cells. The JAK-STAT signaling pathway is involved in a variety of important biological processes, including cell proliferation and apoptosis. To explore the effect of lncRNA *EN-90756* on the JAK-STAT pathway, we examined the levels of JAK1 and STAT3 proteins and their phosphorylation. The results showed that JAK1 and p-JAK1 protein levels were significantly down-regulated in CPB2-induced IPEC-J2 cells after knockdown of lncRNA *EN-90756*. STAT3 and p-STAT3 proteins were barely detected in CPB2-induced IPEC-J2 cells after knockdown of lncRNA *EN-90756* (Figures 6D, E). The results suggested that lncRNA *EN-90756* regulates *MX1* expression and affects JAK-STAT pathway activation in CPB2-induced IPEC-J2 cells.

## 4. Discussion

*Clostridium perfringens* causes enteritis and enterotoxaemia in domestic animals, wildlife and humans, and its virulence is mainly due to its ability to produce toxins. CPB2 toxin is an important pathogenic factor causing necrotizing enteritis in animals. The correlation between neonatal piglet diarrhea and the presence of *C. perfringens* capable of producing CPB2 toxin in their intestine is extremely high (Bueschel et al., 2003). To further investigate the effects of CPB2 toxin on animal diarrhea, CPB2 toxin protein was isolated and purified. CPB2 toxin protein was moderately cytotoxic to human NCM460 intestinal epithelial cells (Zeng et al., 2016); and induced apoptosis and inflammation in porcine small intestinal epithelial cells, impairing the intestinal barrier function (Gao et al., 2020). However, the effects of host lncRNAs in CPB2 toxin-treated IPEC-J2 cells and how they are regulated are unclear. Here, IPEC-J2 cells from the CPB2 and control groups were analyzed using RNA-seq to search for lncRNAs affected by CPB2 toxin and to explore the function of these lncRNAs, which will inform studies on the effects of CPB2 toxin on piglet diarrhea.

Long non-coding RNAs have emerged as important regulators of various biological processes and diseases, interacting with other molecules and thus participating in the regulation of histone modifications, gene transcription, RNA stability, RNA splicing, and transcriptional or translational modifications. LncRNA *PVT1* promotes cancer initiation and progression by acting as a competing endogenous RNA (ceRNA), activating STAT3 signaling or KAT2A acetyltransferase, or interacting with myelocytomatosis oncogene (MYC) (Zhao J. et al., 2018; Jin et al., 2019; Sun et al., 2019). Systemic loss-of-function experiments demonstrate that long intergenic ncRNAs (lincRNAs) are involved in the molecular circuitry of embryonic stem cells and are required for the maintenance of embryonic stem cell pluripotency (Guttman et al., 2011). In recent years, numerous studies related to lncRNAs have been conducted in domestic animals. The 73 up-regulated and 68 down-regulated differentially expressed lncRNAs were identified in placentas from Meishan pigs at the establishment and expansion stages of placental fold development, and these differential lncRNAs were associated primarily with the placental fold development process (Deng et al., 2020). In the present study, we detected 49 significantly differentially expressed lncRNAs between control IPEC-J2 cells and CPB2 treated cells. The target genes of significantly different lncRNA *EN-90756* were mainly associated with defense responses to viruses, suggesting a possible regulatory role of lncRNA *EN-90756* in CPB2-treated cells.

Aberrant lncRNA expression is implicated in the pathogenesis of many diseases. Highly expressed lncRNA *LINC00707* mediates a range of biological functions in humans, including cell proliferation, apoptosis, metastasis, invasion, cell cycle arrest, inflammation, and even osteogenic differentiation (Yao et al., 2022). In present study, the expression of lncRNA *EN-90756* was significantly up-regulated with increasing treatment time of CPB2 toxin and reached its highest value at 24 h. EdU and cell viability assays showed that knockdown of lncRNA *EN-90756* inhibited the proliferation of CPB2-treated IPEC-J2 cells.

The ΔΨm plays a crucial role in many biological processes and is highly correlated with apoptosis (Tian et al., 2019). In epithelial cancer cells, ΔΨm is often abnormally higher than that in normal cells, which is associated with increased invasiveness of cancer cells *in vitro* and their increased metastatic potential *in vivo* (Begum et al., 2021). Instability of the ΔΨm leads to early reperfusion arrhythmias and systolic dysfunction (Ashok et al., 2020). Treatment with isoliquiritigenin increased apoptosis in human bladder cancer T24 cells in a concentration-dependent manner and caused a decrease in the ΔΨm in a time-dependent manner (Si et al., 2017). It has been reported that ΔΨm decreased in CPB2-treated IPEC-J2 cells (Luo et al., 2020). Here, we noted that knockdown of lncRNA *EN-90756* resulted in a significant decrease in the ΔΨm in IPEC-J2 cells, implying that the cells entered an early apoptotic phase. Initiator caspases (such as Caspase 8) are involved in early apoptotic signaling, and once Caspase 8 is activated, it can immediately initiate downstream effector caspases (e.g., Caspase 3) to promote apoptosis (O’Donnell et al., 2011; Willson, 2019; Zhaojun et al., 2022). We found that CPB2-induced the mRNA expression of apoptotic proteins *Caspase 3* and *Caspase 8* in IPEC-J2 cells, which was significantly increased after lncRNA *EN-90756* inhibition. Meanwhile, flow cytometry showed that down-regulation of lncRNA *EN-90756* expression promoted apoptosis in IPEC-J2 cells. In summary, knockdown of lncRNA *EN-90756* promoted apoptosis in CPB2-induced IPEC-J2 cells.

The localization of lncRNAs is closely related to their mode of regulation. Although most lncRNAs are transcribed by RNA polymerase II (Pol II) and have polyadenylate tails and m7G cap structures, they are processed and spliced less efficiently and exhibit significant cytosolic localization (Derrien et al., 2012; Tian and Manley, 2017; Guo et al., 2020). When localized in the nucleus, lncRNAs can interact with a variety of molecules such as DNA, RNA, and proteins to regulate chromosome structure and function, or regulate gene transcription in *cis* or *trans*, affecting mRNA splicing, stabilization, and translation (Fanucchi et al., 2019; Statello et al., 2020). LncRNAs localized in the cytoplasm are mainly involved in the regulation of gene expression in *trans* at the post-transcriptional level, such as the regulation of mRNA translation and degradation, or in the regulation of intracellular signaling pathways (Hartford and Lal, 2020). Special organelle-localized lncRNAs are involved in organelle function and metabolic regulation, such as the mitochondrial oxidative response and homeostatic balance (Mercer et al., 2011; Carlevaro-Fita et al., 2016; Noh et al., 2016). FISH analysis showed that lncRNA *EN-90756* was distributed in both the nucleus and cytoplasm, but mainly in the cytoplasm, suggesting that lncRNA *EN-90756* may be involved in post-transcriptional regulation. LncRNA *TINCR* regulates the stability of KRT80 by binding to STAU1 protein, thereby affecting epidermal cell differentiation (Kretz et al., 2013). The lncRNA AS Uchl1 controls the translation of Uchl1 *via* an embedded SINEB2 repeat sequence (Carrieri et al., 2012, 2015). No neighboring genes were found in the vicinity of lncRNA En-90756, suggesting that lncRNA En-90756 may function by regulating distal target genes. To further investigate the regulatory role of lncRNA EN-90756 in IPEC-J2 cells, we examined significantly differentially expressed target genes. We noted a significant up-regulation of MX1 expression after lncRNA EN-90756 knockdown. *MX1* is an interferon-stimulated gene that plays a role in the defense of mammalian cells against influenza A and other viruses by repressing viral gene transcription (Pavlovic et al., 1992; Holzinger et al., 2007; Raftery and Stevenson, 2017; Spitaels et al., 2019). MX1 represents a key component of the mouse innate immune system that protects mice from the highly lethal human H5N1 influenza virus (Tumpey et al., 2007). LncRNA *IVRPIE* promoted the host immune response against influenza A virus by regulating the expression of interferon β1 and MX1 (Zhao et al., 2020). These results suggest that lncRNA *EN-90756* might influence the immune response of IPEC-J2 cells by regulating MX1 expression, providing clues for disease resistance breeding and disease treatment in piglet diarrhea.

Previous studies confirmed that the JAK/STAT pathway plays an important role in mediating cell fate such as apoptosis, differentiation, and proliferation (Muoz-Cánoves et al., 2013; Richard and Stephens, 2014; O’Shea et al., 2015; Jia et al., 2016). The JAK family of receptor-associated tyrosine kinases act as primary transducers of multiple cytokine receptors and mediate a variety of their effects through the phosphorylation of STAT transcription factors (Derecka et al., 2012; Lee et al., 2016). LncRNA *FEZF1-AS1* promotes proliferation and inhibits apoptosis in ovarian cancer through activation of the JAK-STAT3 pathway (Zhao X. et al., 2018). Crizotinib could promote cell apoptosis of human lung cancer cell line H2228 by regulating the expression of JAK and STAT proteins in the JAK-STAT signaling pathway (Lu et al., 2018). LncRNA *MEG3* inhibited the migration of oral squamous cell carcinoma cells and promoted apoptosis by regulating the JAK-STAT pathway (Tan et al., 2019). Previous studies have shown that CPB2 toxin treatment of IPEC-J2 cells inhibits cell proliferation, promotes apoptosis, and accelerates the inflammatory response (Gao et al., 2020; Luo et al., 2020; Yang et al., 2022). Here, lncRNA *EN-90756* was able to alleviate CPB2 toxin-induced apoptosis and promote cell proliferation in IPEC-J2 cells. In addition, the JAK-STAT signaling pathway was regulated by lncRNA *EN-90756*, as inferred from the expression levels of related proteins. The above results suggested that lncRNA *EN-90756* might regulate the proliferation and apoptosis of IPEC-J2 cells by affecting the JAK-STAT pathway. However, the regulation of lncRNA is complex and the specific mechanism of regulation of JAK-STAT by lncRNA *EN-90756* needs to be further investigated.

In conclusion, this study identified differentially expressed lncRNAs in the CPB2 and control groups of IPEC-J2 cells. In addition, we found that CPB2 toxin treatment promoted significant differential lncRNA *EN-90756* expression up-regulation. Inhibition of lncRNA *EN-90756* suppressed the proliferation of CPB2-treated IPEC-J2 cells and promoted apoptosis. Mechanistically, lncRNA *EN-90756* might affect the antiviral ability of IPEC-J2 cells by regulating the expression of MX1. Meanwhile, lncRNA *EN-90756* might regulate CPB2-induced cell proliferation and apoptosis by affecting the JAK-STAT signaling pathway. These findings provide novel perspectives and directions for further exploration of the regulatory mechanisms of lncRNAs toward CPB2 toxin-induced diarrhea in piglets.

## Data availability statement

The datasets presented in this study can be found in online repositories. The names of the repository/repositories and accession number(s) can be found below: https://www.ncbi.nlm.nih.gov/, PRJNA749943.

## Author contributions

SG and JY: conceived and designed the study. JY, JZ, QY, XH, ZY, PW, and XG: experimental investigation and data analysis. JY, JL, NL, and YG: data curation. JY: writing—original draft. QY: writing—review and editing. All authors have read and agreed to the published version of the manuscript.

## Footnotes

1
www.bioinformatics.babraham.ac.uk/projects/fastqc/

2
ccb.jhu.edu/software/hisat2/

3
ccb.jhu.edu/software/stringtie/
